# Conceptualizing the substrates and sequelae of decreased sound tolerance as a developmental cascade: A pilot study

**DOI:** 10.1016/j.heares.2025.109472

**Published:** 2025-11-13

**Authors:** Ava Schwartz, Grace Pulliam, Jacob I. Feldman, Kacie Dunham-Carr, S. Madison Clark, Kelsea McClurkin, Carissa J. Cascio, Bahar Keçeli-Kaysılı, Tiffany Woynaroski

**Affiliations:** a Neuroscience Undergraduate Program, Vanderbilt University, Nashville, TN, USA; b Neuroscience Graduate Program, Vanderbilt University, Nashville, TN, USA; c Department of Hearing & Speech Sciences, Vanderbilt University, Nashville, TN, USA; d Frist Center for Autism and Innovation, Vanderbilt University, Nashville, TN, USA; e Vanderbilt Brain Institute, Vanderbilt University, Nashville, TN, USA; f Department of Hearing and Speech Sciences, Vanderbilt University Medical Center, Nashville, TN, USA; g Current affiliation: Rosamund Stone Zander Translational Neuroscience Center & Department of Neurology, Boston Children’s Hospital, Brookline, MA, USA; h Vanderbilt Kennedy Center, Vanderbilt University Medical Center, Nashville, TN, USA; i Department of Psychiatry, Vanderbilt University Medical Center, Nashville, TN, USA; j Current affiliations: Department of Psychology and Life Span Institute, University of Kansas, Lawrence, KS, USA; k Department of Communication Sciences and Disorders, John A. Burns School of Medicine, University of Hawaii at Manoa, Honolulu, HI, USA

**Keywords:** Gamma power, EEG, Sensory hyperresponsiveness, Decreased sound tolerance, Hyperacusis, Anxiety, Baby siblings

## Abstract

A growing body of research has shown that decreased sound tolerance (DST) is highly prevalent and impacts the mental health of affected individuals. Recent work has shown this is especially true for autistic individuals. The extant literature has been limited, however, by a focus on DST relatively late in life. Consequently, at present we know little about when and how DST emerges and produces cascading effects on mental health. In this pilot study, we prospectively followed infants at high likelihood for autism, and thus hypothetically for DST, based on their status as younger siblings of autistic children (Sibs-Autism) and infants at lower, general population-level likelihood for these conditions (Sibs-NA) to determine whether (a) DST symptomatology differs based on autism likelihood status and/or diagnostic outcome; (b) indices of early resting brain states, specifically gamma power, predict sensory hyperresponsiveness and DST; (c) sensory hyperresponsiveness predicts DST symptomatology; (d) DST symptomatology predicts anxiety; and (e) if the aforementioned associations vary by familial likelihood for autism or later autism status. Preliminary results indicate that DST symptoms are elevated in Sibs-Autism, particularly those who go on to receive a diagnosis of autism, relative to Sibs-NA. Gamma power is not significantly associated with later sensory hyperresponsiveness or DST, but hyperresponsiveness is associated with later DST, which is associated with later anxiety in Sibs-Autism.

Autism is a neurodevelopmental condition that is characterized by differences in social communication and by the presence of restricted and repetitive patterns of behavior, interests, and activities (American Psychiatric Association [APA], 2022) and that has long-term effects on social, academic, and vocational outcomes of affected children (Billstedt et al., 2007; Chamak and Bonniau, 2016; Mason et al., 2021). Sensory differences are also highly prevalent in autism (e.g., Ben-Sasson et al., 2019; Kirby et al., 2022), and are now considered diagnostically relevant (APA, 2022).

## The cascading effects theory of sensory differences

**Fig. 1.**
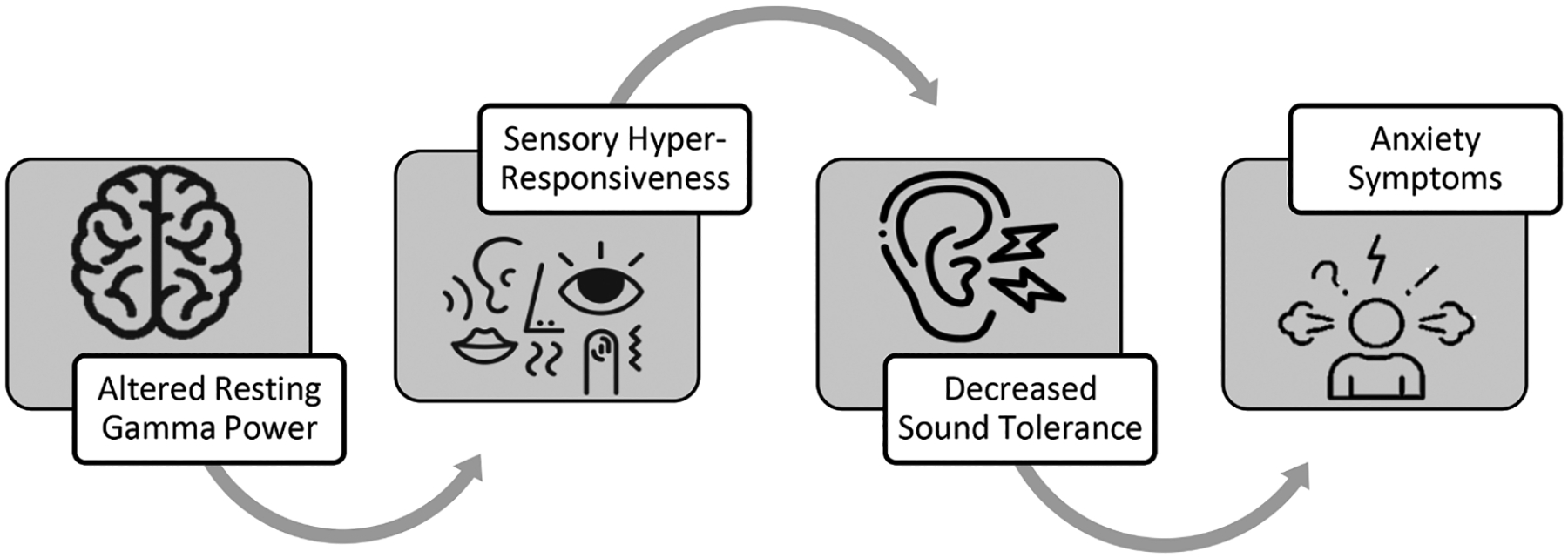
Conceptual model of decreased sound tolerance (DST) as a developmental cascade.

The cascading effects theory posits that sensory differences may emerge early in life from alterations in resting brain states that “cascade” onto development across domains, which may ultimately yield a constellation of differences in development, including the core and commonly co-occurring features of autism (e.g., Cascio et al., 2016; Simon and Wallace, 2016). The present study employs this cascading effects framework in an attempt to elucidate the predictors and consequences of one sensory difference that is frequently observed in autism—decreased sound tolerance (DST). See Fig. 1 for an overview of the conceptual framework motivating this work, which is summarized in the following sections.

## Decreased sound tolerance in autism

DST is defined as an inability to tolerate everyday sounds (Jastreboff and Jastreboff, 2015; Williams, He, et al., 2021). This sensory difference is highly prevalent in autism, with 50–70 % of autistic individuals estimated to experience DST symptoms at some point in their lives (Williams, Suzman, and Woynaroski, 2021). Many autistic individuals report severe discomfort due to their DST symptoms, and DST has been reported to be one of the most disabling sensory features of autism (Scheerer et al., 2022; Stefanelli et al., 2020). Despite the high prevalence of DST in the autistic population, it remains a largely understudied phenomenon, particularly early in life.

## Rationale for prospectively following infant siblings of autistic children (Sibs-Autism)

Measuring early sensory differences pertaining to autism can be challenging because autism cannot always be reliably diagnosed in the first few years of life (e.g., Turner and Stone, 2007; Woolfenden et al., 2012). As such, infant siblings of autistic children (i.e., Sibs-Autism) are now often prospectively studied to explore early developmental differences relevant to autism (Rogers, 2009; Szatmari et al., 2016). Sibs-Autism are approximately 8–10 times more likely to be diagnosed with autism as compared to infants at population-level likelihood for autism (i.e., infants who have no autistic older siblings [Sibs-NA]; Maenner et al., 2023; Ozonoff et al., 2011). Hypothetically, this increased likelihood for a future diagnosis of autism additionally places Sibs-Autism at high risk for DST. In the present study, we test this hypothesis for the first time, additionally evaluating predictors and sequelae of DST in Sibs-Autism that are motivated by theory and past research.

### Sensory hyperresponsiveness as a putative predictor of DST in sibs-autism

One construct that theory and research suggest may predict DST is generalized sensory hyperresponsiveness (also referred to by other terms such as hyperresponsivity, hyperreactivity, sensitivity, and over-responsivity; Andermane et al., 2023; He et al., 2023). Hyper-responsiveness is marked by exaggerated responding to sensory stimuli across various sensory modalities (e.g., vision, olfaction, somatosensation, gustation, audition, and proprioception; Ben-Sasson et al., 2009). Hyperresponsiveness is prevalent in young autistic children and has additionally been observed early in life in Sibs-Autism (Baranek et al., 2007; Wolff et al., 2019). While hyperresponsiveness has been correlated with concurrent DST in children and adults (e.g., Andermane et al., 2023; Rinaldi et al., 2023; Wu et al., 2014), this will be the first study to test predictive links between hyperresponsiveness and later DST in Sibs-Autism and Sibs-NA.

## Gamma power as a potential neural substrate of sensory hyperresponsiveness and DST

Differences in brain oscillatory activity may predict hyper-responsiveness and DST, particularly oscillatory power in the gamma frequency band. Oscillatory power indexes the synchronous rhythms of cortical activity that can be measured on the scalp via electroencephalography (EEG), with resting EEG power reflecting oscillatory activity in the absence of an active task or “at rest” across a range of frequency bands with varying functional differences (Başar et al., 2001; Wang, 2010). Spectral analyses are often conducted to derive absolute power (i. e., the integral or average of all power values within a frequency band), which quantifies the magnitude of oscillations across the range of relevant frequencies within a band/s (Saby and Marshall, 2012).

Gamma power is of particular interest due to its role in the synchronization of responses to sensory stimuli across distributed neural networks. Gamma oscillatory activity has been associated with the binding of perceptual features in early sensory responses and has been repeatedly identified as an index of imbalance in neural network excitation and inhibition, which has been implicated in some models of autism (Herrmann et al., 2010; Rubenstein and Merzenich, 2003; Whittington et al., 2011). Differences in gamma power and related indices of excitatory/inhibitory imbalance (e.g., thalamic concentration of gamma-aminobutyric acid [GABA] as measured via magnetic resonance spectroscopy) have been associated with disruptions in sensory processing and hyperresponsiveness across numerous sensory modalities (e.g., Simon and Wallace, 2016; Wood et al., 2021), as well as correlated with sensory gating and theorized to influence responses to auditory stimuli in young children on the autism spectrum (Orekhova et al., 2008). Prior work has also documented alterations in resting gamma power in Sibs-Autism, specifically those who go on to receive a diagnosis, within the first year of life (Gabard-Durnam et al., 2019).

## Anxiety as a likely consequence of DST

A large and growing extant literature suggests that DST is also associated with anxiety. Such links have been observed across children and adults without diagnoses of autism (e.g., Guzick et al., 2023; Rinaldi et al., 2022; Rosenthal et al., 2022; Siepsiak et al., 2023; Smit et al., 2021) and in several studies of autistic adults (Landon et al., 2016; Scheerer et al., 2024; Williams, 2024). However, the research conducted to date has relied almost entirely on retrospective, cross-sectional, or concurrent correlational designs in older children and adults; thus, additional research is necessary to test whether DST early in life predicts later anxiety across clinical and at-risk populations (Poulsen et al., 2024; Rodrigues, 2023; Williams, 2024). No prior study to our knowledge has tested whether DST symptoms predict subsequent anxiety in Sibs-Autism, who are additionally at elevated risk of anxiety symptoms that may manifest within the early childhood period (Shivers et al., 2013). Elucidating such links may facilitate earlier detection and intervention for emerging DST and anxiety symptomatology. Implementing interventions earlier has the potential to improve social, academic, and emotional functioning later in life in this population (Neil and Christensen, 2009).

## Purpose and research questions

The present study, therefore, intended to fill gaps in the current literature in several ways. Specifically, we evaluated whether differences in DST symptomatology were detectable between Sibs-Autism and Sibs-NA by 3 years of age, considering future diagnostic outcomes. Additionally, we examined the extent to which: (a) resting gamma power at 12–18 months of age relates to sensory hyperresponsiveness as measured at approximately 2 years of age and DST as measured at 3 years of age; (b) hyperresponsiveness at 2 years of age relates to DST as measured at 3 years of age; and (c) DST as measured at 3 years of age relates to subsequent anxiety symptomatology at 5–8 years of age. Our research questions were as follows:

- Does DST symptomatology differ based on autism likelihood and/or later diagnostic status by 3 years of age? We hypothesized that DST symptoms would be increased in Sibs-Autism relative to Sibs-NA, especially for the subgroup of Sibs-Autism that went on to receive an autism diagnosis.
- What are the developmental substrates and sequalae of DST? We hypothesized that lower gamma power at 12–18 months of age would be associated with greater hyperresponsiveness at approximately 2 years of age and DST at 3 years of age, that greater hyperresponsiveness at 2 years of age would be associated with greater DST symptomatology at 3 years of age, and that greater DST symptomatology at 3 years of age would be associated with greater anxiety at 5–8 years of age.
- Are any of the aforementioned associations moderated by (a) familial likelihood or (b) later diagnostic status? We hypothesized that some of the associations of interest may vary according to likelihood status and/or later diagnostic outcome, such that relations may be stronger in Sibs-Autism versus Sibs-NA, perhaps particularly in the subgroup of Sibs-Autism that received an autism diagnosis.

## Methods

### Participants

Participants in this study included 40 infants and toddlers (20 Sibs-Autism and 20 Sibs-NA) who were drawn from a larger longitudinal study at Vanderbilt University Medical Center and matched at the group level on both chronological age and biological sex (see Table 1). Of these participants, 8 Sibs-Autism (40 %) went on to receive an autism diagnosis (Sibs-Autism-dx), and 12 Sibs-Autism (60 %) did not (Sibs-Autism-nodx). No Sibs-NA were later diagnosed with autism. Eligibility criteria included: (a) chronological age 12–18 months at study entry, (b) full term birth (gestation > 37 weeks), (c) no concomitant genetic disorders, (d) no known adverse neurological history, (e) no history of or present concerns regarding sensorineural hearing loss or vision impairment; (f) primarily English-speaking household, and (g) at least one older sibling either diagnosed with autism (Sibs-Autism) or without an autism diagnosis (Sibs-NA). For Sibs-Autism, the diagnostic status of older sibling/s was confirmed by a licensed clinician using the Autism Diagnostic Observation Schedule, Second Edition (ADOS-2; Lord et al., 2012) and clinical interview, either within our laboratory or by record review. In the Sibs-NA group, the diagnostic status of older sibling/s was verified with the Social Communication Questionnaire (SCQ; Rutter et al., 2003), on which the older sibling(s) must have scored below the threshold for autism concern (i.e., < 15).

### Overview of study design

Participants were first seen in the laboratory for Time 1 study visits between 12–18 months of age, at which time resting gamma power was measured via eyes-open resting state EEG. Hyperresponsiveness was measured at Time 2 study visits, when participants were between 21–27 months of age. DST was measured at Time 3, after each participant’s 3rd birthday. Finally, anxiety was measured at Time 4, when participants were between 5–8 years of age. See Table 2 for an overview of study constructs, measures, variables, and timepoints.

### Measures

#### Time 1 – resting gamma power

Eyes-open resting state EEG was recorded when infants were 12–18 months old (i.e., at Time 1). Participants sat quietly on their parents’ laps in a sound- and light-attenuated psychophysiology laboratory. Parents were instructed to help their child sit as still as possible and watch a muted video that involves simple moving shapes (i.e., ‘Baby Einstein’, Kids II, Inc.). EEG data were collected from 128 electrodes using a NetAmp 400 amplifier and Geodesic Sensor Net (Electrical Geodesics Inc.). The target was 10 min of resting state EEG data collection; an average of 605 ± 29 s of data were recorded per participant (range 599 – 803 s). Data were acquired at a sampling rate of 1000 Hz, referenced to the vertex (Cz), and online filtered from 0.1 to 100 Hz. Data were exported and processed further using EEGLAB (Delorme and Makeig, 2004), down sampled to 250 Hz, and band-pass filtered with a zero-phase finite impulse response filter between 0.5 and 50 Hz.

Epochs 2 s long with 50 % overlap were extracted, baseline corrected to the mean and visually inspected for artifacts and bad channels. Bad channels (3.31 ± 2.26) were removed. Residual artifacts were corrected with independent component analysis. Data were then re-referenced to the average, and removed channels were interpolated. Peripheral electrodes (26 total) were excluded from all analyses due to high levels of artifact contamination and interpolation. A total of 195 ± 96 epochs were retained per participant (range 61– 392). This did not vary by sibling group (199 ± 85 epochs retained in the Sibs-Autism group; 191 ± 104 epochs retained in the Sibs-NA group; *p* = .80). No participants were excluded from analyses due to low initial EEG recording lengths and/or artifacts that resulted in fewer than 60 epochs of usable EEG data.

EEG epochs were transformed using a zero padded fast Fourier transform (0.061 Hz resolution) after application of a Hann window. Whole scalp power spectra were calculated by averaging amplitude across all electrodes and squaring to confirm the presence of distinct bands. Amplitude values were averaged across all remaining electrodes, squared to power, and log10 transformed. Absolute power in the gamma band was extracted between 30–50 Hz.

#### Time 2 – sensory hyperresponsiveness

Sensory hyperresponsiveness was measured 9 months later (i.e., at Time 2, when participants were 21–27 months old) via two caregiver questionnaires: the Sensory Experiences Questionnaire (SEQ; Baranek et al., 2006) and the Infant/Toddler Sensory Profile Caregiver Questionnaire (SP; Dunn, 1999). The SEQ characterizes sensory responsiveness across a range of sensory modalities and social and nonsocial contexts. It yields an index of mean hyperresponsiveness that was used in analyses. The SP similarly characterizes early sensory processing. It yields two indices of sensory hyperresponsiveness, Sensory Sensitivity and Sensation Avoiding, which were used in analyses after being reflected, such that higher scores indicated increased hyperresponsiveness across measures.

#### Time 3 – DST

DST was measured via the SP and SEQ around participants’ third birthday (i.e., at Time 3) by summing the scores from the SEQ and (reflected) SP items tapping auditory hyperresponsiveness at this timepoint (see Table 3). Our prior work provides support for the psychometrics of these measures to tap modality-specific sensory response patterns (Williams et al., 2023). The scores derived additionally had excellent reliability in the present sample (Cronbach’s *a* = 0.836).

#### Time 4 – anxiety

Anxiety was measured for all participants when they were 5–8 years old (i.e., at Time 4) using the Parent-Rated Anxiety Scale for Youth with Autism Spectrum Disorder (PRAS-ASD; Scahill, 2019). The PRAS-ASD is a previously developed and validated caregiver report intended to measure anxiety in children with autism between 5–17 years of age (see Maddox et al., 2020). The total score was used in analyses to tap overall anxiety symptomatology in this sample.

#### Across timepoints – diagnostic outcomes

Diagnostic status was determined for all Sibs-Autism and Sibs-NA via comprehensive developmental evaluations, including the ADOS-2 and a clinical interview with a licensed clinician on our research team, across all follow-up visits (i.e., across Times 2–4).

### Analytic plan

Prior to conducting analyses, variables were evaluated for normality, specifically for skewness > |1.0| and kurtosis > |3.0|. A square root transformation was applied to the PRAS total score to correct for positive skew. Subsequently, missing data (ranging from 0 to 20 % across variables derived for use in analyses) were imputed using the *missForest* package (Stekhoven and Bühlmann, 2012) in RStudio (R Core Team, 2023). Component variables tapping hyperresponsiveness, including the (reflected) SP Sensory Avoidance Score, (reflected) SP Sensory Sensitivity Score, and SEQ Hyperresponsiveness Mean Score, were z-transformed and averaged to create a hyperresponsiveness aggregate score for use in analyses. All component variables used to tap hyper-responsiveness were sufficiently intercorrelated to warrant aggregation following z-score transformation (i.e., > 0.4; Feldman et al., 2024; Rushton et al., 1983; see Table 4). An ANOVA was run to evaluate differences in DST between Sibs-Autism-dx, Sibs-Autism-nodx, and Sibs-NA. A series of regression analyses was then carried out to test relations of interest. Likelihood group and diagnostic outcome were considered as moderators, and Cook’s D was utilized to monitor for undue influence across modeling. Discrete points with Cook’s D greater than 4/*n* were flagged as potential outliers. The results summarized below were all robust to excluding these discrete data points.

### Sensitivity analysis

Sensitivity analyses indicated that, with *n* = 40 and α = 0.05, we were powered at 80 % to detect effects of interest that were moderate to large in magnitude in this pilot study. The planned ANOVA was powered (1-*β* = 0.80) to detect an omnibus effect of group with *f*^*2*^ > 0.26. Regressions were powered (1-*β* = 0.80) to detect associations with *f*^*2*^ > 0.21 and 0.30 with one predictor and three predictors in the model, respectively (in tests of zero-order and moderated relations).

## Results

### RQ1: group differences in time 3 DST

**Fig. 2.**
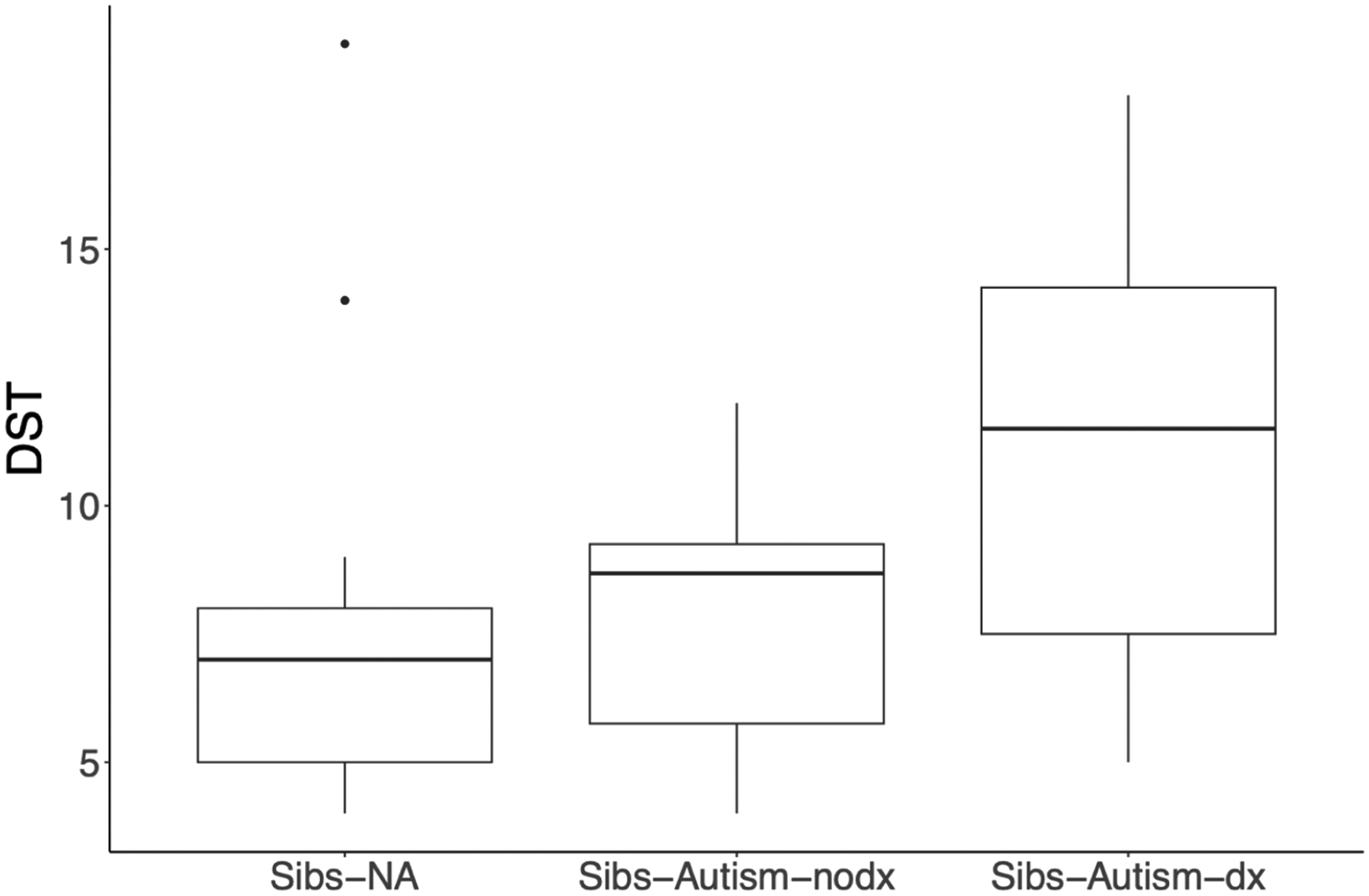
Time 3 decreased sound tolerance symptomatology by likelihood group and diagnostic status *Note*. Decreased sound tolerance (DST) symptomology, as indexed by an aggregate of selected item-level data from the Sensory Experiences Questionnaire (SEQ; Baranek et al., 2006) and Infant/Toddler sensory profile caregiver questionnaire (SP; Dunn, 1999) administered at Time 3 significantly varied according to group, *F* (2, 37) = 4.217, *p* = .047, *η*^*2*^ = 0.19. Sibs-NA = younger siblings of non-autistic children; Sibs-Autism-dx = younger siblings of autistic children who received a diagnosis of autism; Sibs-Autism-nodx = younger siblings of autistic children who did not receive a diagnosis of autism.

The between-group difference in Time 3 DST was statistically significant, *F*(2,37) = 4.217, *p* = .047, *η*^*2*^ = *0*.19 (see Fig. 2). DST symptomatology was highest for Sibs-Autism-dx (*M* = 11.125, *SD* = 4.611), followed by Sibs-Autism-nodx (*M* = 7.954, *SD* = 2.717), followed by Sibs-NA (*M* = 7.583, *SD* = 3.924). The effect sizes for the between-group differences for Sibs-Autism-dx relative to Sibs-Autism-nodx and Sibs-NA were large in magnitude (Cohen’s *d* = 0.889 and 0.858, respectively). The difference between Sibs-Autism-nodx and Sibs-NA was negligible (*d* = 0.101).

### RQ2: developmental cascade of DST

**Fig. 3.**
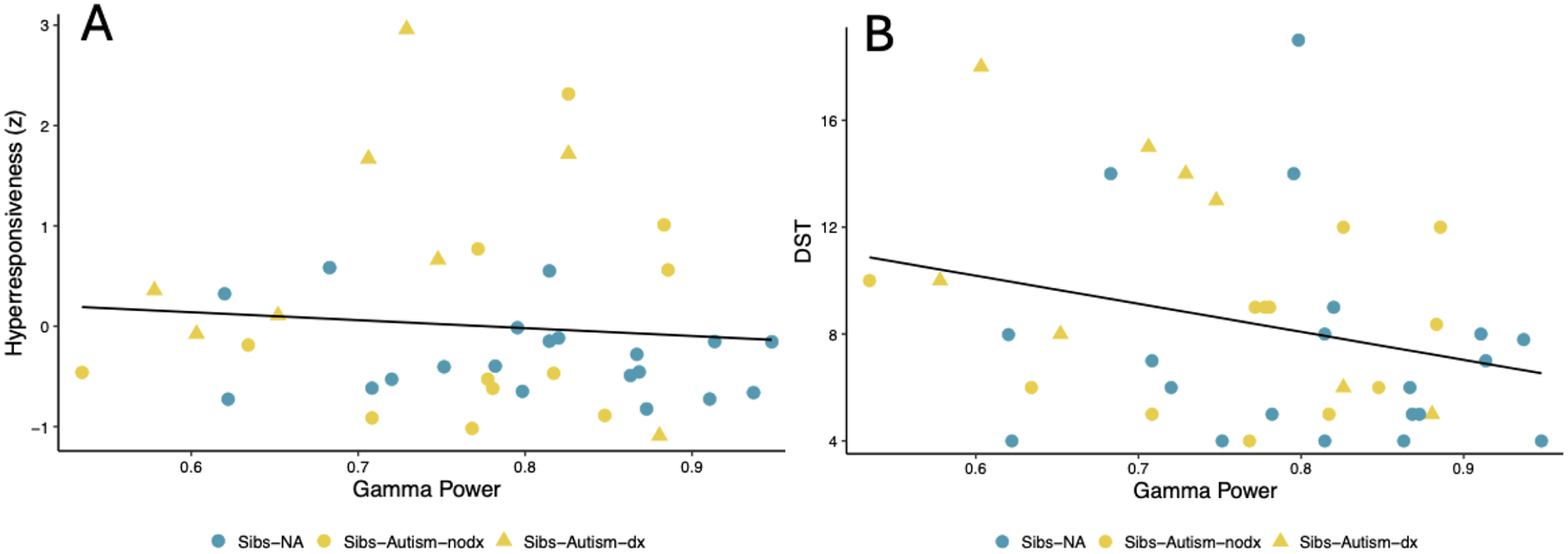
Scatterplot depicting the association between time 1 gamma power and time 2 hyperresponsiveness and time 3 decreased sound tolerance *Note*. The log of absolute gamma power as measured by resting state EEG at Time 1 was not significantly associated with sensory hyperresponsiveness at Time 2 as derived from relevant component variables from the Sensory Experiences Questionnaire (SEQ; Baranek et al., 2006) and Infant/Toddler Sensory Profile Caregiver Questionnaire (SP; Dunn, 1999) at Time 2 (Fig. 3A), or with Decreased Sound Tolerance (DST), as indexed by selected items tapping DST from the SEQ and SP as collected at Time 3 (Fig. 3B). These associations did not vary by likelihood or diagnostic status. Sibs-NA = younger siblings of non-autistic children; Sibs-Autism-dx = younger siblings of autistic children who received a diagnosis of autism; Sibs-Autism-nodx = younger siblings of autistic children who did not receive a diagnosis of autism.

**Fig. 4.**
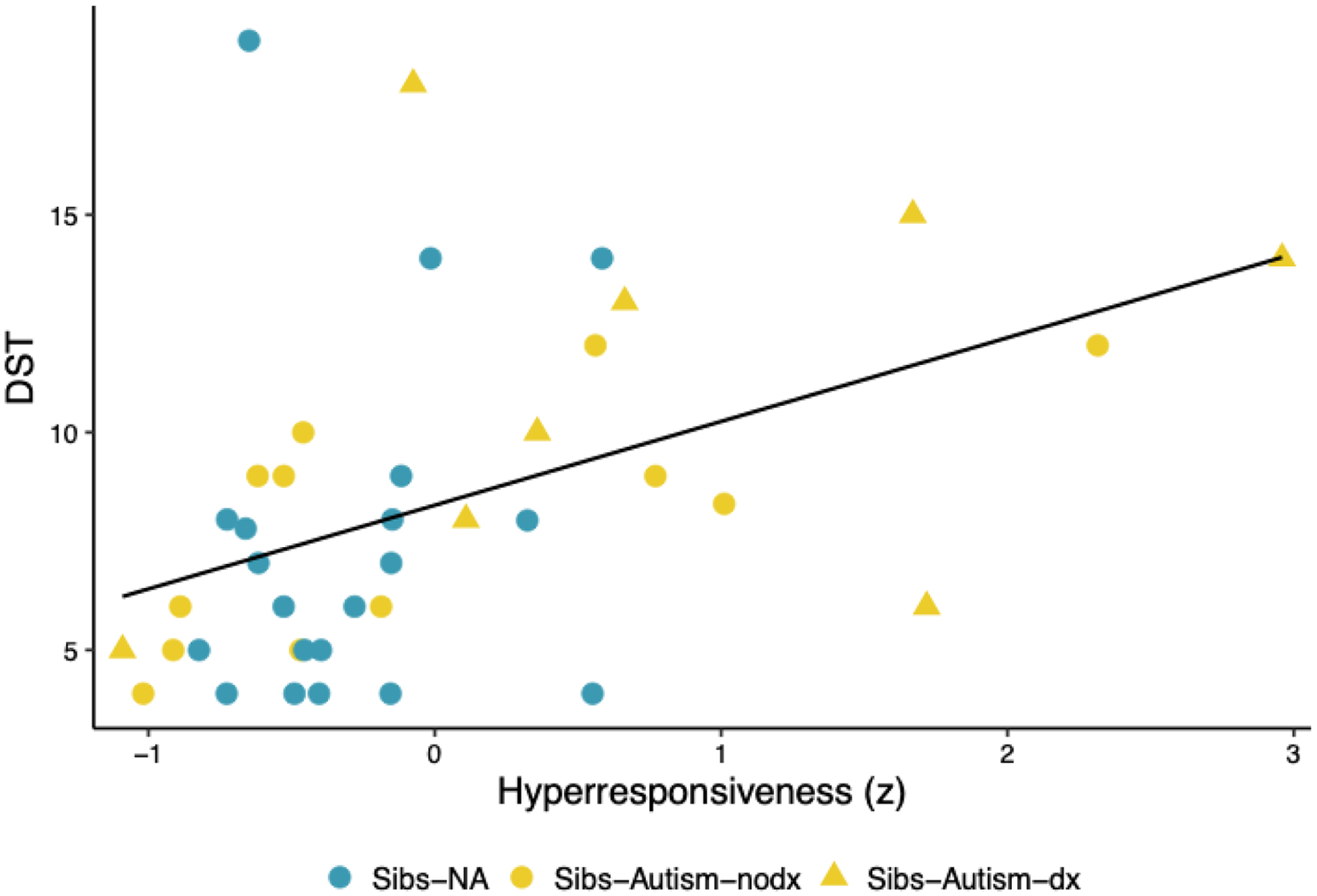
Scatterplot Depicting the Association Between Time 2 Hyperresponsiveness and Time 3 Decreased Sound Tolerance *Note*. Hyperresponsiveness, as derived from relevant component variables from the Sensory Experiences Questionnaire (SEQ; Baranek et al., 2006) and Infant/Toddler Sensory Profile Caregiver Questionnaire (SP; Dunn, 1999) at Time 2, was significantly associated with Decreased Sound Tolerance (DST), as indexed by items tapping DST from the SEQ and SP as collected at Time 3. This association did not vary according to likelihood status or diagnostic outcome. Sibs-NA = younger siblings of non-autistic children; Sibs-Autism-dx = younger siblings of autistic children who received a diagnosis of autism; Sibs-Autism-nodx = younger siblings of autistic children who did not receive a diagnosis of autism.

Time 1 gamma power was not significantly associated with Time 2 sensory hyperresponsiveness (*ß* = −0.176, *p* = .279) or Time 3 DST (*ß* = −0.274, *p* = .087), though these (small) associations were in the anticipated direction (See Fig. 3A and 3B). Time 2 hyperresponsiveness was significantly and positively associated with Time 3 DST, with a moderate effect size (*ß* = 0.393, *p* = .012; see Fig. 4). Further, Time 3 DST was significantly and positively associated with Time 4 anxiety, with a moderate effect size across groups (*ß* = 0.428, *p* = .006).

### RQ3: moderation by group

**Fig. 5.**
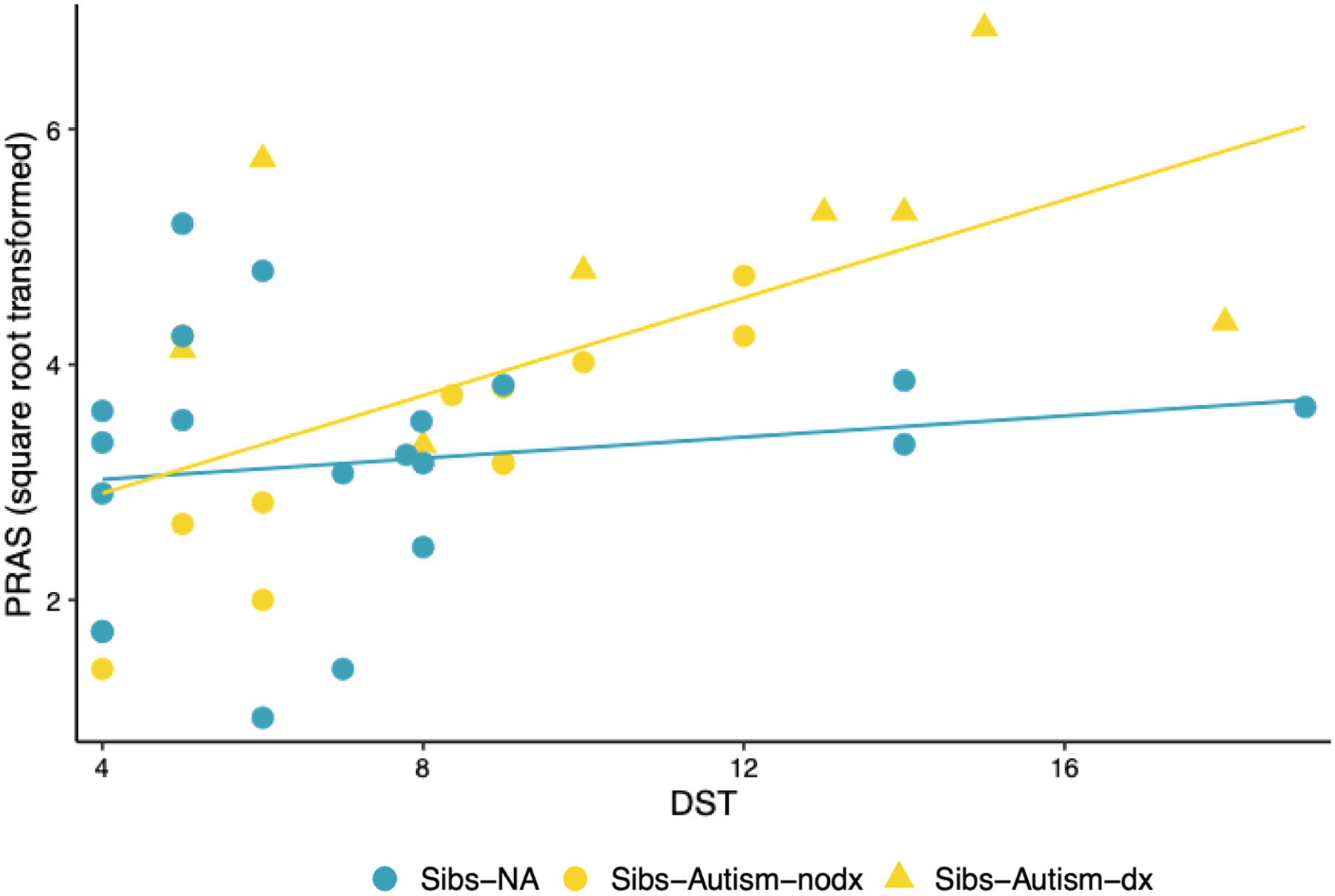
Scatterplot Depicting the Association Between Time 3 Decreased Sound Tolerance and Time 4 Anxiety *Note*. Decreased Sound Tolerance (DST), as indexed by item-level data from the Sensory Experiences Questionnaire (SEQ; Baranek et al., 2006) and Infant/Toddler Sensory Profile Caregiver Questionnaire (SP; Dunn, 1999) as collected at Time 3, was significantly associated with anxiety at Time 4 as indexed by the (square-root transformed) total score for the Parent-Rated Anxiety Scale for Autism Spectrum Disorder (PRAS; Scahill, 2019). This association varied according to likelihood status, such that it was significant in Sibs-Autism (inclusive of those who did receive a diagnosis [Sibs-Autism-dx; depicted in yellow triangles] and those who did not receive a diagnosis [Sibs-Autism-nodx; depicted in yellow dots]) but was not significant in Sibs-NA.

The relation between Time 3 DST and Time 4 anxiety was significantly moderated by likelihood group (*p* value for the product term in the regression model testing the moderated effect = 0.039; see Fig. 5), but not by diagnostic status (*p* value for the product term in the regression model testing the moderated effect = 0.150). Post-hoc tests indicated that this association was significant and large in magnitude in Sibs-Autism (*r* = *0*.548, *p* = .007), but was not significant and negligible in magnitude in Sibs-NA (*r* = 0.081, *p* = .736). No other relations of interest were significantly moderated by either likelihood group or diagnostic status.

## Discussion

This investigation was the first to our knowledge to study DST early in life in the younger siblings of autistic and non-autistic children. Our results provide new insights into the presence, predictors, and sequelae of DST symptomatology in these populations. These findings have important implications for theory, research, and future clinical practice.

### DST symptoms are detectable early in life in some infants

DST significantly differed according to autism likelihood and diagnostic outcome, such that children at a high likelihood for autism (i.e., Sibs-Autism) who went on to receive a diagnosis themselves (i.e., Sibs-Autism-dx) tended to present with higher DST symptomatology as compared to both children at an increased likelihood who did not receive an autism diagnosis and children without a family history of autism at 3 years of age. This finding supports the notion that it may be possible to detect DST earlier in life, perhaps allowing for early intervention on DST symptoms, particularly in Sibs-autism who are on a different developmental trajectory. Notably, some participants within the Sibs-NA group also presented with elevated DST at 3 years old, suggesting that such symptoms may emerge relatively early in life for at least some children at low familial likelihood for autism as well.

### Sensory hyperresponsiveness precedes and predicts DST symptomatology

Our results additionally indicate that sensory hyperresponsiveness as measured around 2 years of age (i.e., between 21–27 months old) predicts DST at 3 years old. This finding suggests that early features of hyperresponsiveness across sensory modalities may serve as a signal for future sound intolerance. This association was, notably, not moderated by likelihood or diagnostic status, indicating that these associations are present across young children at both high and low familial likelihood for autism regardless of their diagnostic outcomes.

### DST symptomatology predicts subsequent anxiety

Our findings further indicate that there is a significant association between DST and subsequent anxiety. However, this association varied according to likelihood group, suggesting that sound intolerance around 3 years of age predicts anxiety symptoms within the 5–8-year-old window only in children with a familial predisposition for autism. If this is the case, then intervening upon early differences in sound tolerance, may prevent or mitigate the emergence of anxiety later in childhood in this high likelihood population.

### Failure to detect significant associations for gamma power and later DST

We failed to detect significant relations between resting gamma power as measured at 12–18 months and later sensory hyper-responsiveness and DST. Though we observed trends in the anticipated direction, the relations were small in magnitude and not statistically significant. Admittedly, this study was underpowered to detect effects of this size. Thus, larger-scale studies are needed to evaluate the association between gamma power, hyperresponsiveness, and DST. Such studies are warranted, given emerging research suggesting causal links between gamma and hyperresponsiveness, and specifically DST. In the context of such research, investigators may also consider measuring more localized gamma (e.g., within auditory cortex), deriving other resting state indices (e.g., of connectivity or complexity within the gamma band), using different paradigms to tap evoked gamma power, and/or testing whether effects of interest vary according to other participant characteristics (e.g., age and sex) that have been implicated as moderators in the extant literature (e.g., Clayton et al., 2024; Dede et al., 2025; McGill et al., 2023).

### Implications for theory, research, and clinical practice

These findings lend increasing empirical support for the theory that early sensory alterations may cascade onto downstream traits that are commonly observed in children with or at high likelihood for autism, and more specifically for the recent proposal that DST may arise from generalized hyperresponsiveness and induce anxiety (Andermane et al., 2023; Cascio et al., 2016). The present results further suggest that employing the “baby sibs” model—prospectively following infant and toddler siblings of autistic children—is promising for helping us to determine precisely when and how sound intolerance emerges and impacts broader development early in life. Such research will likely be beneficial from a clinical standpoint, for facilitating our collective efforts to identify and intervene upon sensory differences like DST before they have fully manifested and affected mental health.

### Strengths, limitations, and future directions

This study represents a crucial first step in elucidating how and when DST develops in children with or at high likelihood for autism. However, the investigation is not without limitations. First, this pilot was limited by its small and rather homogenous sample. Across groups, almost all infants were White and non-Hispanic, limiting the generalizability of our findings to broader populations. Additionally, specific DST conditions that commonly co-occur with autism were not comprehensively evaluated in this initial study, and the measures used to assess the superordinate DST construct here likely tapped early symptoms of hyperacusis more so than misophonia or phonophobia. Thus, our findings are limited in furthering our understanding of the development of specific DST disorders, as well as any relations between them. Finally, we leveraged individual questions from sensory questionnaires tapping behavioral responses to auditory stimuli to create the DST aggregate; thus, there may be some shared variance across our hyperresponsiveness and DST aggregates. In a newly funded, supplemental study, our team will build upon these results by adding another timepoint (Time 5) wherein audiologic profiles will be comprehensively characterized and DST conditions, including misophonia and hyperacusis, will be more thoroughly assessed in a larger sample of Sibs-Autism and Sibs-NA, permitting more sophisticated tests of our theorized developmental cascade and advancing us towards more effective early identification and intervention of sound tolerance issues across children at high and low familial likelihood for autism.

## Figures and Tables

**Table 1:** Summary of participant characteristics at study entry according to group.

	Sibs-Autism (*n* = 20) *M* (*SD*)	Sibs-NA (*n* = 20) *M* (*SD*)
Time 1 Age (Months)	14.73 (2.14)	14.73 (2.26)
Time 1 MSEL ELC*	89.33 (13.71)	99.15 (9.30)
Sex Assigned at Birth	*n* (%)	*n* (%)
Male	11 (55 %)	11 (55 %)
Female	9 (45 %)	9 (45 %)
Race	*n* (%)	*n* (%)
Black/African American	0 (0 %)	1 (5 %)
White	20 (100 %)	17 (85 %)
Multiple Races	0 (0 %)	2 (10 %)
Ethnicity	*n* (%)	*n* (%)
Hispanic or Latino	0 (0 %)	1 (5 %)
Not Hispanic or Latino	20 (100 %)	19 (95 %)

*Note*. Time 1 = Entry to the longitudinal study, MSEL ELC = Early Learning Composite score from the Mullen Scales of Early Learning (Mullen, 1996).

* Denotes groups significantly differed, *p* < .01.

**Table 2:** Summary of key study constructs, measures, and variables.

Construct	Measure/s	Variable(s)	Role Per RQ	Time Point
Likelihood Group	Demographic form, ADOS-2, SCQ	Infant sibling of (a) autistic child/ren, as confirmed via the ADOS-2, or (b) only non-autistic child/ren, as confirmed via score below threshold for autism concern (i.e., score of 15) on the SCQ	IV/RQ1 Putative moderator/RQ3	Time 1
Diagnostic Outcome	ADOS-2, interview	Clinical best estimate (dichotomized) based on administration of ADOS-2 and clinical interview	IV/RQ1 Putative moderator/RQ3	Times 2–4
Gamma Power	RSEEG	Log absolute power in the band from 30–50 Hz	Predictor/RQ2&3	Time 1
Hyper-responsiveness	SP SEQ	Average of z scores for: (a)Sensory Sensitivity score from SP (reflected) (b)Sensation Avoiding score from SP (reflected) (c)Mean hyperresponsiveness score from SEQ	Predictor & Outcome (DV)/RQ2&3	Time 2
Decreased Sound Tolerance	SP SEQ	Sum of scores for items tapping auditory hyperresponsiveness on the SP (8, 11) and SEQ (1, 6)	DV/RQ1 Predictor & Outcome (DV)/RQ2&3	Time 3
Anxiety	PRAS-ASD	Total score	Outcome(DV)/RQ2&3	Time 4

*Note*. RQ = research question, DV = dependent variable, IV = independent variable, ADOS-2 = Autism Diagnostic Observation Schedule, Second Edition (Lord et al., 2012), SCQ = Social Communication Questionnaire (Rutter et al., 2003), SP = Infant/Toddler Sensory Profile Caregiver Questionnaire (Dunn, 1999), SEQ = Sensory Experiences Questionnaire (Baranek et al., 2006), RSEEG eyes-open resting state electroencephalography, PRAS-ASD = Parent-Rated Anxiety Scale for Autism Spectrum Disorder (Scahill et al., 2019).

**Table 3:** Summary of items used to create decreased sound tolerance aggregate.

Questionnaire	Item Number	Content
SP	8	“My child startles easily at sound, compared to other children the same age.”
SP	11	“My child tries to escape from noisy environments.”
SEQ	1	“Does your child react sensitively or startle easily to unexpected or loud sounds? (For example: cover ears when hearing a vacuum, baby cry, door close, etc.)”
SEQ	6	“Does your child show distress (startles, covers ears, etc.) during loud conversations or singing?”

*Note*. SP = Infant/Toddler Sensory Profile Caregiver Questionnaire (Dunn, 1999), SEQ = Sensory Experiences Questionnaire (Baranek et al., 2006).

**Table 4:** Intercorrelations of component variables used to generate hyperresponsiveness aggregate.

Component Variable	1	2	3
1.SP Sensory Avoidance Score	—		
2.SP Sensory Sensitivity Subscale Score	.665***	—	
3.SEQ Hyperresponsiveness Mean Score	.766***	.867***	—

*Note*. SP = Infant/Toddler Sensory Profile Caregiver Questionnaire (Dunn, 1999), SEQ = Sensory Experiences Questionnaire (Baranek et al., 2006). Note that SP scores were reflected to ensure that higher scores represented higher hyperresponsiveness across measures.

*** *p* < .001.
